# A hyper-temporal remote sensing protocol for high-resolution mapping of ecological sites

**DOI:** 10.1371/journal.pone.0175201

**Published:** 2017-04-17

**Authors:** Jonathan J. Maynard, Jason W. Karl

**Affiliations:** USDA-ARS, Jornada Experimental Range, MSC 3JER, New Mexico State University, Las Cruces, NM, United States of America; Universita degli Studi di Trento, ITALY

## Abstract

Ecological site classification has emerged as a highly effective land management framework, but its utility at a regional scale has been limited due to the spatial ambiguity of ecological site locations in the U.S. or the absence of ecological site maps in other regions of the world. In response to these shortcomings, this study evaluated the use of hyper-temporal remote sensing (i.e., hundreds of images) for high spatial resolution mapping of ecological sites. We posit that hyper-temporal remote sensing can provide novel insights into the spatial variability of ecological sites by quantifying the temporal response of land surface spectral properties. This temporal response provides a spectral ‘fingerprint’ of the soil-vegetation-climate relationship which is central to the concept of ecological sites. Consequently, the main objective of this study was to predict the spatial distribution of ecological sites in a semi-arid rangeland using a 28-year time series of normalized difference vegetation index from Landsat TM 5 data and modeled using support vector machine classification. Results from this study show that support vector machine classification using hyper-temporal remote sensing imagery was effective in modeling ecological site classes, with a 62% correct classification. These results were compared to Gridded Soil Survey Geographic database and expert delineated maps of ecological sites which had a 51 and 89% correct classification, respectively. An analysis of the effects of ecological state on ecological site misclassifications revealed that sites in degraded states (e.g., shrub-dominated/shrubland and bare/annuals) had a higher rate of misclassification due to their close spectral similarity with other ecological sites. This study identified three important factors that need to be addressed to improve future model predictions: 1) sampling designs need to fully represent the range of both within class (i.e., states) and between class (i.e., ecological sites) spectral variability through time, 2) field sampling protocols that accurately characterize key soil properties (e.g., texture, depth) need to be adopted, and 3) additional environmental covariates (e.g. terrain attributes) need to be evaluated that may help further differentiate sites with similar spectral signals. Finally, the proposed hyper-temporal remote sensing framework may provide a standardized approach to evaluate and test our ecological site concepts through examining differences in vegetation dynamics in response to climatic variability and other drivers of land-use change. Results from this study demonstrate the efficacy of the hyper-temporal remote sensing approach for high resolution mapping of ecological sites, and highlights its utility in terms of reduced cost and time investment relative to traditional manual mapping approaches.

## Introduction

Continued advancements in geospatial technologies and their application to the science of global change is providing an increasingly accurate and resolved depiction of the widespread degradation occurring across the Earth’s terrestrial ecosystems [1,2]. These technological advancements have brought into clearer focus the growing need for broad-scale assessments of landscape condition that provide a quantitative, systematic, and iterative (i.e., adaptive management) framework for informing land management actions. Concurrently, advances in the field of ecology have provided a more refined understanding of ecosystem dynamics and the biotic and abiotic factors that impart resistance and/or resilience to degradation from natural and anthropogenic drivers of change. This has resulted in a theoretical shift from a climax-based perspective where ecosystems are tightly coupled and internally regulated [3–5], to an alternative stable state perspective that incorporates ecosystem heterogeneity, resilience, thresholds, non-linearity, and feedbacks to external forcing [6–10]. From this theoretical shift, the concept of ecological potential, that is, the ability of a given land type to sustainably support various ecosystem services (e.g., agricultural yield, forage quality/quantity, water quality, etc.), has emerged as a guiding principle in many land-use management systems [11–13]. Growing insight into the primary controls on ecosystem potential (soil, topography, climate) coupled with advances in remote sensing technologies, is providing new opportunities to create quantitative, systematic, and spatially-explicit modeling frameworks for the prediction of landscape units with similar ecological potential.

Among the ecological potential-based classification systems, ecological site descriptions (ESDs) and associated state-and-transition models (STM) have been characterized as the world’s most extensive land management framework, with a management footprint of nearly 370 million ha in the United States [14]. ESDs and STMs have been developed with the intent of guiding land management decisions at a national level, allowing the coordination and prioritization of conservation funding for the management and restoration of terrestrial ecosystem services [15]. In the U.S., ecological sites (ESs) represent soil and climate-based land classes that differ in their potential vegetation communities (i.e., reference state) and the natural and anthropogenic processes that result in transitions to alternative stable states [16–18]. Thus, ESs represent static land classes that maintain a set range of ecological potential, constrained by their inherent soil properties and climate. While the vegetation composition on a given ES can change in response to disturbance resulting in potential state changes, its inherent potential does not change. For example, the reference state of the Loamy ES class is a grassland, but when highly degraded (e.g., drought, over grazing) can shift to a shrub dominated state. The patterns and mechanisms of state transitions are formally described for each ES in a STM. STMs are synthetic descriptions of the dynamics of plant communities and associated changes in ecosystem services and management needs within specific ESs [19–21]. STMs use diagrams and data-supported narratives to describe these dynamics and provide evidence for the causes. Recently the concept of generalized ecological state [13,22] has emerged as a way to generalize the description of ecological states within STMs based on similarities in their initial reference state (e.g., historical grassland vs historical savanna) and the range of drivers that initiate state transitions. For example, in southern New Mexico, USA, grasslands exhibit similar trajectories of change, moving from a historical grassland (maximum productivity, historically dominant species), to an altered grassland (decreased productivity, erosion, invasive species), to a shrub-invaded grassland (low productivity, erosion, shrub expansion), and finally to a shrubland state (near complete loss of grasses, erosion/soil degradation) as the site is increasingly degraded (see Table 1 for description of general states). This provides a standardized framework to describe the potential range of ecological variability that can exist within each ES.

**Table 1:** Generalized ecological state classes found within the focal study region of the USDA ARS Jornada Experimental Range.

State Code	General State	Concept for general state†
1	Grassland	Site near maximum productivity, populated with full complement historically dominant grass species.
2	Altered Grassland/Savanna	Site often exhibits reduced total annual and/or forage production. If historically dominant species are present, these are fragmented and/or subdominant to less-palatable, grazing-tolerant or ruderal species. Evidence of soil erosion.
3	Shrub/Tree Savanna	Site near maximum productivity, populated with full complement historically dominant shrub/tree and grass species.
4	Shrub/tree-invaded	Woody plants expanding into perennial grassland become dominant over or codominant with grazing-tolerant grasses. Remnant patches of historically dominant grass species may persist in woody plant interspaces suggesting that competitive exclusion is incomplete and/ or soil degradation infrequent. Soil redistribution to shrub patches apparent. Reduced grass connectivity leads to reduced fire occurrence.
5	Shrub/tree-dominated	Soil is redistributed to and biological activity is centered beneath expanding woody plants. Scattered perennial grass cover (<10%) exists as relict patches in shrub interspaces. Grazing tolerant or ruderal grass species occur under shrubs. Evidence of interspace erosion/soil degradation, resource retention is low. Facilitation between shrubs and grasses sustains remaining grasses.
6	Shrubland/ woodland	Near complete loss of perennial grasses in shrub interspaces. Perennial grass species may occur as isolated plants. Woody plants are dominant. Extensive evidence of interspace erosion/soil degradation, resource retention is very low.
7	Bare/annuals	Perennial species absent or occur as isolated relict plants. Erosion extensive. Resource retention is very low.

†Adapted from Bestelmeyer et al. 2009 and Steele et al., 2012.

In its current implementation, the mapping of ESs in the U.S. is correlative in nature; that is, ES designations are assigned to existing soil series based on relevant soil properties, existing or potential vegetation composition, geomorphology, and climate [15]. However, given the scale at which soils are mapped in the U.S. (i.e., Soil Survey Geographic (SSURGO) database Order 3: ~1:24,000 scale), a single soil map unit may contain multiple soil series (i.e., soil map unit components) whose spatial location within the map unit is unknown, and thus the precise location of associated ESs is also unknown. Also, criteria for the spatial delineation of SSURGO soil map units is often different than those used to separate landscapes according to ecological potential concepts such as ESs [23,24]. This presents many challenges from a management perspective, as it often results in a spatial and thematic mismatch in the data available to land managers. Consequently, there is a growing need to extend the utility of ESs by developing a quantitative and systematic methodology for mapping ESs at the spatial scales needed for implementing management actions and/or restoration measures. In addition, such a framework should be scalable and thus capable of being implemented at both regional and national scales.

In response to these shortcomings, several studies have employed remote sensing and geospatial techniques to predict the spatial distribution of ESs [22–27]. Accurate prediction of ESs requires spatially explicit knowledge of the soil, geomorphic, and vegetation characteristics that control the ecological potential of a given landscape unit. While prior remote sensing efforts have demonstrated the efficacy of delineating distinct vegetation types using multi-spectral imagery [28,29], accurate detection of differences in soil types has been less successful [30,31]. Current efforts to map ESs on a landscape scale require extensive field work in order to properly identify soil properties associated with a given ES. Consequently, the majority of efforts to map ESs have relied upon prior generalized soil maps and correlated ESs (e.g., SSURGO map units) as a primary input into modeling efforts. This approach is problematic in heterogeneous landscapes where the actual location of ESs is obscured within soil map units containing multiple soil components. Also, the creation of spatially explicit maps of ESs is complicated by the fact that ESs can exist in different ecological states. Thus the accurate mapping of ESs requires not only the ability to identify and delineate the ES’s reference state, but also all other possible stable states.

From a remote sensing perspective, areas identified as having similar spectral properties in a single image (e.g., similarities in surface greenness due to similar plant functional types) may experience vastly different temporal responses to variation in precipitation or temperature due to underlying differences in soil properties [32]. While soil properties are used to classify ESs, it is the effect of those properties on soil processes and their response to climate and management drivers that ultimately results in differences between ESs [18]. Consequently, ES classification is a system that represents ecosystem dynamics; therefore, the accurate classification and delineation of ESs requires approaches that can adequately quantify these temporal dynamics.

**Fig 1:**
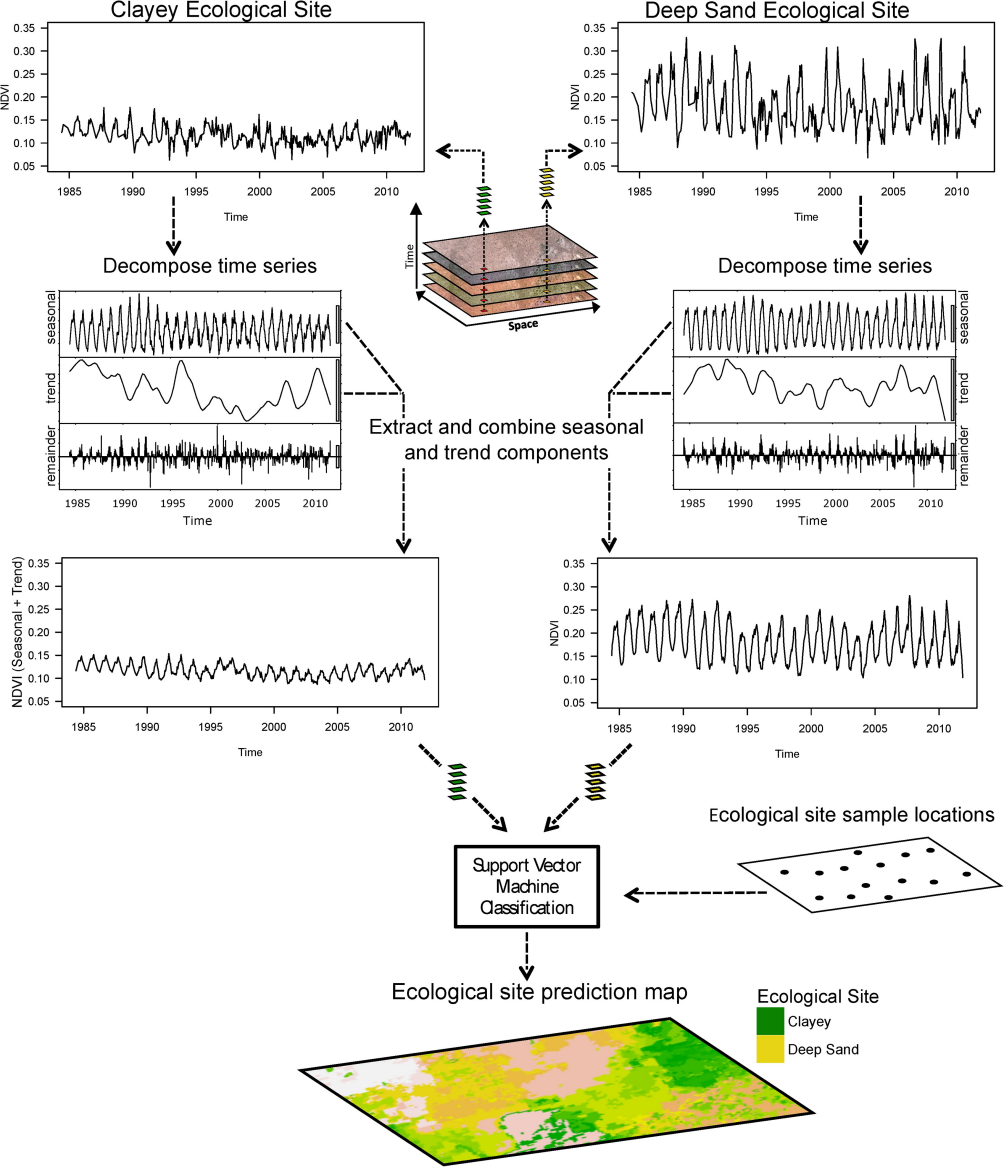
Conceptual workflow illustrating the hyper-temporal remote sensing modeling framework. Modeling steps include the compilation of Landsat TM 5 NDVI layers from 1984–2011, the decomposition and extraction of the seasonal and trend components of the time series for each pixel stack, and the integration of the modified time series and sampled field data into a support vector machine classification model to produce ecological site class prediction maps.

Recent work [31] has demonstrated the utility of using hyper-temporal (i.e., hundreds of images) remotely sensed normalized difference vegetation index (NDVI) to predict soil properties in a semi-arid ecosystem. In this work they showed that temporal variability in vegetation spectra, as driven by soil and climate feedbacks, is an important predictor of soil variability and can be used to characterize a ‘fingerprint’ of the soil-vegetation-climate relationship. Given the tight coupling between vegetation, soils and climate in differentiating unique ESs, we hypothesized that hyper-temporal remote sensing would also be effective in predicting ESs in semi-arid ecosystems. This hypothesis was premised on the understanding that soils modulate the response of vegetation to climatic variability, in particular climatic extremes (i.e., physiological response to prolonged drought or elevated rainfall). Through quantifying temporal variability in land surface spectral properties (e.g., NDVI) we can gain greater insight into the spatial distribution of soil properties that regulate vegetation response (e.g., soil texture, soil depth, soil minerology). Consequently, the hyper-temporal remote sensing approach may provide valuable insight into the spatial distribution of ESs (and possibly states) by identifying areas with similar vegetation communities (i.e., similar phenology), as well as similar soil types (i.e., similar spectral response to climatic variability) (Fig 1, see Methods below for details).

The main objective of this study was to evaluate the hyper-temporal remote sensing (RS) modeling framework for predicting ESs in a 15,900 ha area within the USDA ARS Jornada Experimental Range (JER). To test our proposed framework, we used a 28-year time series of NDVI from Landsat TM 5 data to predict the spatial distribution of ESs using support vector machine (SVM) classification. Modeled results were compared to Gridded Soil Survey Geographic (gSSURGO) database and expert delineated maps of ESs, as well as evaluated within the context of precipitation variability using the standardized precipitation index (SPI). Specific objectives were to: (i) evaluate the use of SVM classification for predicting ES classes using hyper-temporal Landsat NDVI, (ii) compare SVM model results to gSSURGO and an expert delineated map of ESs, (iii) evaluate the effects of ecological state on ES model misclassification, and (iv) examine the relationship between precipitation and NDVI spectral variability.

## Methods

### Study area

**Fig 2:**
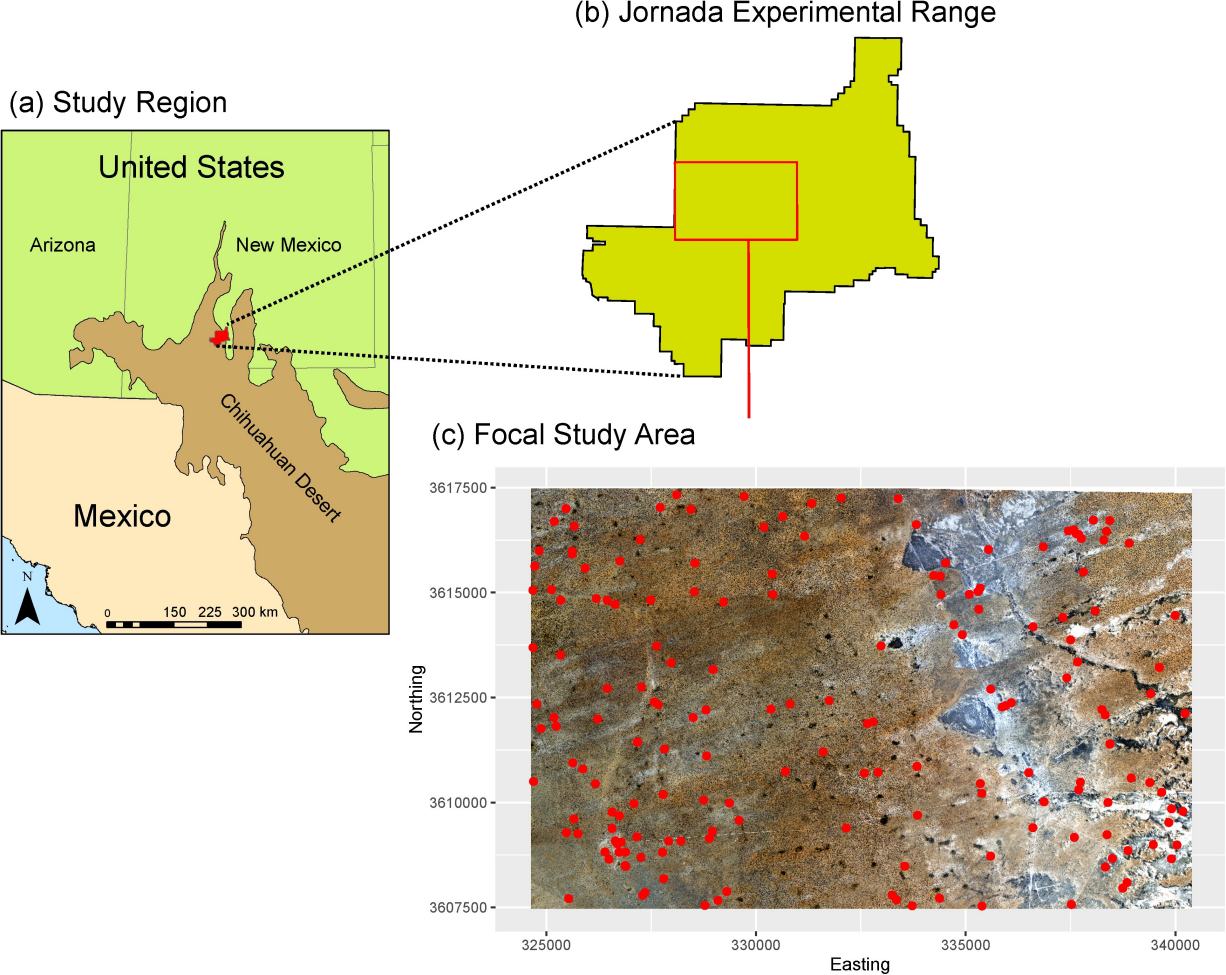
Location of the study area. (a) Study region and site location in southwestern NM, USA; (b) boundary of the Jornada Long-term Ecological Research site with delineated location of focal study area; and (c) focal study area underlain by 1-m National Agriculture Imagery Program (NAIP) imagery and overlain with 176 sampling locations (red circles).

The study was conducted at the JER in southern New Mexico, USA (32.5°N, 106.45°W). The climate in the study area is arid to semiarid with mean annual precipitation of 25 cm over the past 30 years, with the majority of rainfall occurring between 1 July and 1 October during the summer monsoon. Precipitation during the summer monsoons occurs in fast, high-intensity convective rainfall events. During winter months (Nov.-Feb.) perennial deciduous species are dormant and vegetative biomass is at a minimum. In spring, C3 shrubs begin to leaf out (i.e., early- to mid-May) followed by C4 grasses in late summer or early fall which marks the period of peak biomass (i.e., mid- to late-September). Mean monthly temperatures range from 6°C in January to 26°C in June, with an annual mean of 15°C. The JER encompasses approximately 100,000 ha of semi-arid/arid grass-shrubland in the northern region of the Chihuahuan desert (Fig 2). The JER is actively grazed with grazing intensities maintained at light to moderate levels throughout the site. The focal study area, located in the central portion of the JER, encompasses approximately 15,900 ha. The study area was chosen due to its high concentration of field sampling points and diversity of ESs and states (Fig 2). The study area has an east to west elevation gradient and is comprised of several geomorphic features; including relict basin floors, piedmont slopes, shrub-coppice dunes, and playas. Dominant ESs within the study area are Clayey, Loamy, Sandy, Shallow sandy, and Deep sand ESs. Additional information on the soils, vegetation, and ecological dynamics for each ES can be found in S1 Table. Despite differences among the ESs, similarities in the sequence and trajectory of state changes can be seen depending on the ESs initial reference state. Table 1 provides descriptions of each generalized state and its designated state code (SC). For example, ESs with a historical grassland (SC1) as their reference state (e.g., Clayey, Loamy, Shallow sandy, Sandy) will experience similar responses to degradative processes, in particular the transition from a grassland (SC1) to a shrubland (SC6) or bare/annuals (SC7) state. Similarly, ESs with a historical savanna (SC3) as their reference state (e.g., Deep Sand) will experience similar transitions between states (i.e., transition from Shrub/Tree Savanna (SC3) to Shrub/tree-dominated (SC5) to Shrubland/woodland (SC6)).

### Modeling framework

The hyper-temporal RS modeling framework consisted of four steps: (1) field data collection, (2) RS image pre-processing, (3) image selection and model building, and (4) model validation and spatial prediction. The modeling framework is presented in Fig 1, illustrating the processing steps at two locations representing different ESs (i.e., Clayey, Deep sand). Image preprocessing consisted of a temporal decomposition of the NDVI time-series, where the seasonal and trend components were extracted and recombined to produce a time-series with a higher signal-to-noise ratio for subsequent modeling. The NDVI values along the filtered time-series at each ES sample location were then evaluated relative to the dependent dataset (i.e., field-verified ESs) using a recursive feature elimination procedure, and significant covariates (i.e., NDVI image dates) retained and used in a SVM classification model to predict ESs. The ecological site prediction model was then used to predict ecological site classes at each 30 m pixel across the study area. All data processing and statistical analysis was done using R software [33].

### Field data collection

This study utilized field data from 176 sample locations collected between 2005 and 2015 over four sampling campaigns. All field sampling for this study was approved by the Jornada Range Research Station, Agricultural Research Service, USDA. All sampling locations were characterized using standard ecological site and state mapping protocols [22], which included: a vegetation survey, soil sampling, and qualitative soil surface assessment [34].

The sampling designs for each of the four datasets were initially created to meet different study objectives, and thus their designs ranged from random sampling, stratified random sampling, and rapid field traverses at targeted sites to verify preliminary ecological site/state designations. Thus, the selected focal area was chosen based on a high density of sample points across the four datasets, as well as representing a diversity of ESs and states.

At each sample location a 20 x 20 m plot was established and ocular estimates of foliar cover were recorded for each plant species within the plot using the Domin-Krajina scale, where: + = few, 1 = few-0.1% cover, 2 = 0.1–1% cover, 3 = 1–5% cover, 4 = 5–10% cover, 5 = 10–25% cover, 6 = 25–33% cover, 7 = 33–50% cover, 8 = 50–75% cover, and 9 = >75% cover [35]. The method of soil sampling differed across the four datasets, including; soil mini-pits (n = 15) (i.e., full profile characterization to 100cm or restrictive layer), soil augering to 100 cm or restrictive layer (n = 66), and selective soil sampling (i.e., only a subset of points were verified with augering or examination of surface soils) (n = 95). Plant species composition and soil profile characteristics were used to assign a U.S. Natural Resources Conservation Service ecological site class to each plot. Qualitative soil surface assessments, in combination with information on dominant and indicator plant species, were used to assign an ecological state class to each plot based on the state-and-transition model associated with its designated ecological site.

From our combined point dataset, five unique ESs were identified, including: Sandy (n = 86), Shallow sandy (n = 16), Deep sand (n = 32), Loamy (n = 37), and Clayey (n = 5). Ecological states consisted of seven classes assigned a numeric value between 1 and 7 (see Table 1 for class descriptions), with the following distribution of ecological states: State 1 = 14, State 2: n = 21, State 3: n = 8, State 4: n = 75, State 5: n = 11, State 6: n = 37, and State 7: n = 10.

### Landsat image acquisition and pre-processing

All available Landsat Thematic Mapper (TM) 5 imagery for the study area were acquired between 1984 and 2011 (16-day frequency), totaling 528 scenes (Path 33/Row 37). This study used the Landsat TM 5 surface reflectance product which is ortho-corrected and radiometrically calibrated to surface reflectance using the LEDAPS algorithm [36]. The CFMASK algorithm [37] was used to mask out all pixels identified as containing clouds, cloud shadow, water, or snow. Missing observations due to QA masking or missing scenes (e.g., Landsat 5 scenes processed through NLAPS), were infilled for each pixel-based time series using linear interpolation. The infilling procedure resulted in a complete 16-day time series from 21 May, 1984 to 8 November, 2011, totaling 628 scenes.

We used NDVI to quantify temporal variability in vegetation dynamics for this study. The Landsat TM 5 surface reflectance product contains a precomputed NDVI layer that is calculated from the red and NIR band values using the standard formula of:

NDVI=(ρNIR−ρRed)/(ρNIR+ρRed) (1)

where ρRed is the spectral reflectance in the red wavelength (Landsat band 3) and ρNIR is the spectral reflectance in the near infrared wavelength (Landsat band 4). NDVI is the most commonly used band ratio in ecological research and has been widely used in rangeland studies with varying levels of success [38–42]. Within our study area NDVI reaches a minimum during mid-winter (Dec.-Jan.) and a maximum in early fall (i.e., mid- to late-September).

A seasonal-trend time series decomposition procedure based on LOESS (local polynomial regression fitting) smoothing was performed on each pixel stack (n = 176,545), decomposing the time series into trend, seasonal, and remainder components [43]. A filtered seasonal-trend time series was then created for each pixel stack by recombining the trend and seasonal components while excluding the remainder component (Fig 1). The remainder component contains both signal noise that was not removed during the prior preprocessing steps and that portion of the original signal that the seasonal and trend models were unable to fit. This small loss of information associated with model fitting was considered acceptable given the increase in signal-to-noise ratio resulting from the filtering procedure.

### Ecological site prediction model

SVM classification was used to model the distribution of ESs across the focal study area using the filtered NDVI time-series values extracted at each sample location as our covariate dataset (n = 628). Although SVM models are highly resistant to non-informative predictors [44], we employed recursive feature elimination (RFE) to identify and remove any redundant or non-informative covariates. RFE is a backward selection algorithm that identifies an optimal subset of predictor covariates through iteratively eliminating the covariate(s) with the lowest importance within the model. Covariates are removed according to a predefined range of limits that set the maximum number of covariates within the model at each iteration. For example, in this study we decreased the allowable number of covariates by ten at each iteration (i.e., i1: 600, i2: 590, i3: 580). Model results from all iterations were evaluated and the model with the highest accuracy was chosen to select the optimal subset of model covariates. The RFE procedure identified 611 RS scenes as significant covariates and ranked them by their level of importance to the SVM model. Thus all scenes were assigned a value ranging from 1 to 611, with 1 corresponding to the most significant scene and 611 corresponding to the least significant scene.

SVM is a supervised classification technique based on statistical learning theory that has gained popularity in the environmental sciences due to its effectiveness as a non-linear classifier in high dimensional spaces [45]. SVM models perform well when trained with sparse and noisy input data and are highly resistant to overfitting, making them highly generalizable which allows for appropriate predictions from out-of-sample data [44]. SVM model development requires the selection of an appropriate kernel and the optimization of hyper-parameters relating to the selected kernel based on a tuning procedure. In this study we used the radial basis kernel which is one of the most commonly used SVM kernels in environmental studies [46]. Optimal estimation of SVM hyper-parameters was performed using a grid-based search approach, where all possible combinations of hyper-parameters were modeled using the training dataset, and hyper-parameters from the model with the highest accuracy were chosen for final model parameterization.

### Model validation

The splitting of observation data into separate training and test sets is a standard approach used to evaluate model performance [44]. However, this approach can result in accuracy metrics with high variance when sample sizes are low or when sample class sizes are highly imbalanced. Consequently, we employed a model validation approach using leave-group-out cross-validation (LGOCV) on our entire dataset (n = 176). The LGOCV procedure is performed by randomly splitting the ecological site observations into training and testing sets, employing a 70%/30% training/testing split. Thus, 70% of observations (training set) were randomly selected for model construction, while the remaining 30% of observations (testing set) were used for model validation, with this process repeated 100 times. LGOCV was used to select optimal tuning parameters, to evaluate the RFE procedure, and to evaluate model performance of our final model of ecological site class. Average accuracy metrics were calculated over the 100 repeated train/test splits. Model predictions and corresponding observation from each of the 100 LGOCV iterations (n = 50/iteration) were compiled and used to create a cross-validated error matrix consisting of 5000 classification values per cross-validated model.

Model performance was assessed using the following metrics: percent correctly classified (PCC), producer’s accuracy, user’s accuracy, and quantity disagreement (QD) and allocation disagreement (AD). PCC is the proportion of test observations that are correctly classified; however, when the sample class distribution is highly unbalanced the PCC may be high due to the over prediction of the largest class [47]. We implemented a weighting routine, where underrepresented classes were given more emphasis in the model, thus preventing the extreme over prediction of the dominant classes (e.g., Sandy) [44]. The models with the highest PCC from the tuning and RFE steps were determined to be the most accurate. Model performance of each individual class within the ecological site classes (e.g., Sandy, Loamy) were assessed with producer’s and user’s accuracy. Producer’s accuracy is a measure of the proportion of sample points correctly classified for a given class relative to the number of observed points of that class, and thus reflects model accuracy in terms of how well the landscape can be mapped. User’s accuracy is a measure of the proportion of sample points correctly classified for a given class relative to the number of predicted points of that class, and thus reflects model accuracy in terms of how reliable the classification map is to the user. The total model disagreement, that is, the difference between the validation and prediction data, can be decomposed into QD and AD [48,49]. Quantity disagreement, QD, represents the amount of difference between the validation and prediction data that is due to a less than perfect match in the proportion of classes. QD is calculated as follows:

$$QD=\frac{1}{2}\sum_{i=1}^{c}\left|p_{i+}-p_{+i}\right|$$ (2)

where *p*_*i*+_ and *p*_+*i*_ represent the row and column totals of the error matrix for the *i*th class for *c* number of classes. Values for QD can range from 0 to 1, where a value of 0 represents perfect agreement in the proportion of coverage for each class between the validation and prediction data. Allocation disagreement, AD, represents the amount of difference between the validation and prediction data that is due to the less than optimal match in the spatial allocation of classes. AD is calculated as follows:

$$AD=[\sum_{i=1}^{c}min(p_{i+},p_{+i})]-C$$ (3)

where *C* is the overall agreement or correct classification. Values for AD can range from 0 to 1, where a value of 0 represents perfect agreement in the spatial allocations for each class between the validation and prediction data.

Although the majority of our training and validation data were collected using different forms of random sampling, our inclusion of data collected using purposive sampling (i.e., rapid field traverses) introduces potential bias in our estimates, resulting in uncertainty as to how well the purposive sampling represents the population. Consequently, our accuracy metrics are representative of our study sample and not necessarily the population within our study area.

### gSSURGO-based mapping of ecological sites

In the U.S., the SSURGO database provides spatial information on the distribution of soil types, delineated by soil map units (SMU). Since SMUs can contain multiple soil components (i.e., soil series), ESs can be linked to one or more components within each SMU, as well as associated with multiple SMUs depending on their similarity with respect to soils, climate and geomorphology. This results in a complex (i.e., many SMUs have a one-to-many correspondence with ESs) and spatially ambiguous representation of ESs. A common approach to deal with this complexity is to represent each SMU polygon by its dominant (i.e., largest areal extent) ecological site. We have chosen to adopt this approach, assigning all SMU classes within our focal study region their dominant ecological site class. For this study we used gSSURGO, a rasterized version the polygon-based SSURGO database [50], with a raster cell resolution of 30 m to correspond to our SVM ES classification based on Landsat imagery.

**Fig 3:**
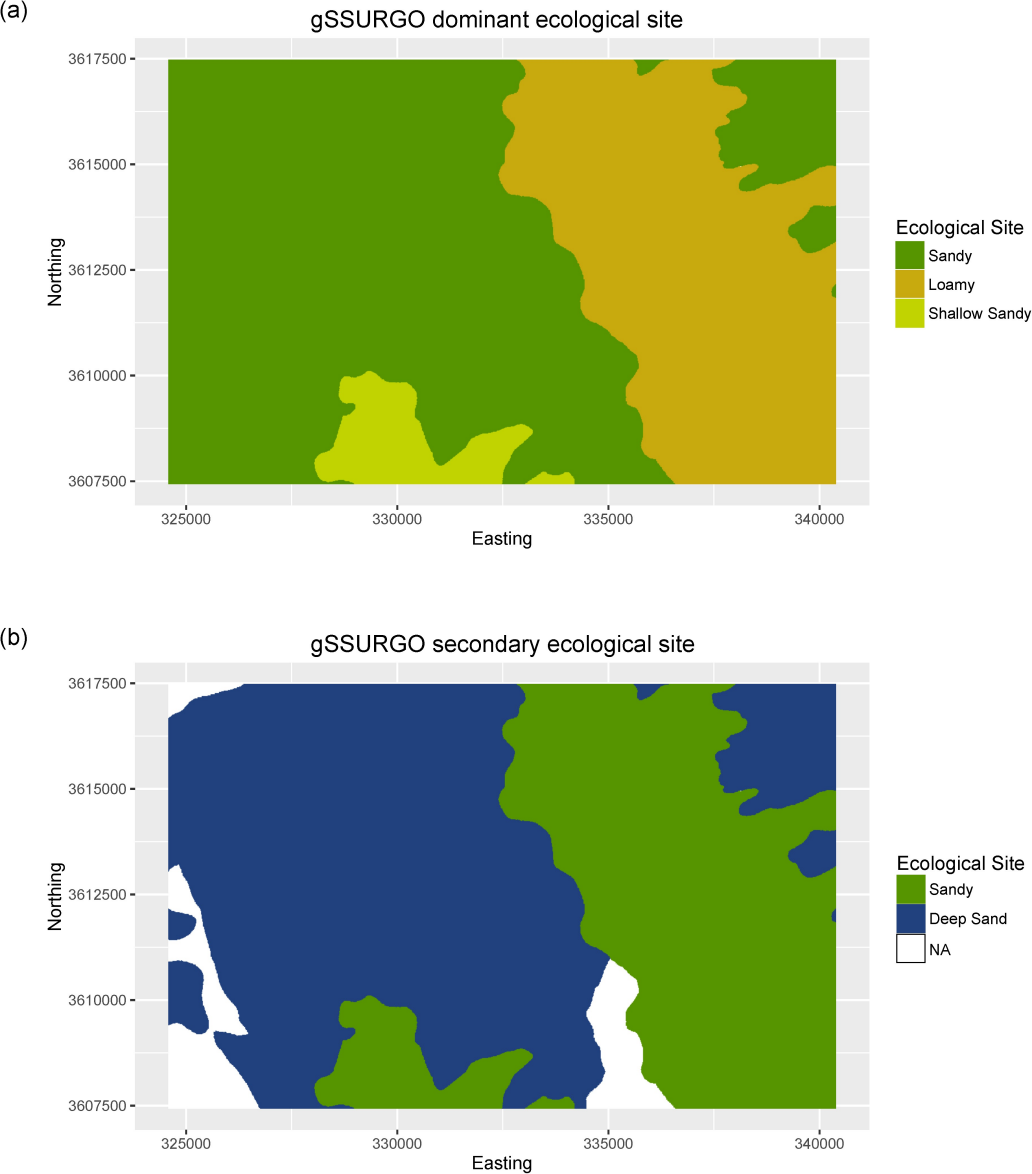
gSSURGO maps of ecological sites. (a) dominant ecological sites, and (b) secondary ecological sites aggregated by dominant condition.

Within our focal study area, gSSURGO identified a total of 7 SMU and 16 SMU components, with each SMU comprised of 2 to 3 components. gSSURGO identified 4 distinct ecological classes (i.e., Loamy, Sandy, Shallow sandy, and Deep sand), with each SMU comprised of either one or two ESs. In many cases multiple soil components within a SMU share the same ecological site classification, and thus we employed the dominant condition approach to spatially represent ecological site classes. All similar ES classes within a SMU were aggregated by their areal extent and the spatially dominant ES class represented (Fig 3A). SMUs that had a less common secondary ES class were also presented (Fig 3B), illustrating the ambiguous spatial complexity of ES within SMUs. Utilizing specified component percentages, Sandy comprised 47% of the study area, Loamy 12%, Deep sand 14%, and Shallow sand 2%, leaving 25% of the study area comprised of inclusions of unspecified soil components and their associated ESs.

### Expert delineation of ecological sites

Expert mapping of ESs is a multi-stepped process that requires a highly trained data analyst. For a detailed description of the methodology, see Steele et al. (2012). In brief, the process begins by classifying SSRUGO soil polygons by their component ESs. This data is then overlain on high resolution orthorectified aerial imagery (e.g., National Agriculture Imagery Program [NAIP]) where the analyst then interprets the aerial imagery to delineate the locations of each ecological site found within a SMU polygon, effectively disaggregating SSURGO map units and creating a finer resolution (i.e., 1:2000–1:5000) ecological site map. At this mapping resolution a minimum mapping unit (MMU) of approximately 1 ha was used, where areas smaller than 1 ha were not mapped. The analysts mapping ESs in this study were skilled photo-interpreters and experts in the ecology and landscapes of southern New Mexico, as well as the STMs that describe ecosystem states.

**Fig 4:**
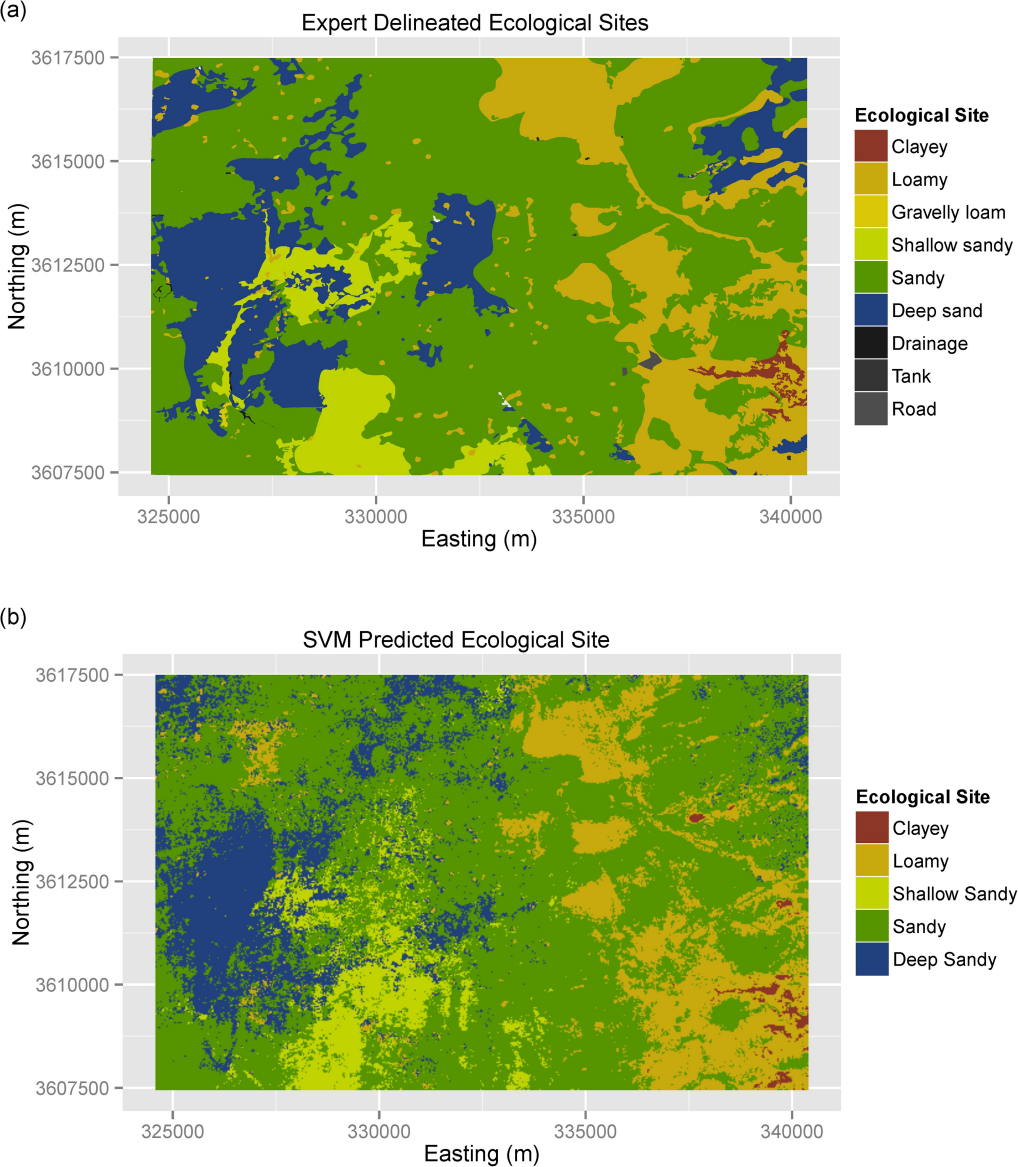
Expert delineated and SVM predicted maps of ecological sites. (a) expert delineated ecological sites and (b) support vector machine predicted ecological sites.

Expert delineation identified nine ESs within the study area, including: Sandy (41% of area), Loamy (31% of area), Deep sand (17% of area), Shallow sandy (7% of area), and Clayey (3% of area). The remaining four ES classes (Gravelly loam, Drainage, Tank and Road) collectively comprised 1% of the study area (Fig 4A). Comparison of the expert map of ES to gSSURGO reveals that gSSURGO effectively captured the dominant ESs present in the study area, as well as a very coarse representation of their spatial locations (Fig 3). In contrast, the expert delineated map provided a detailed representation of the spatial distribution of ESs across the study area.

### Standardized precipitation index

The standardized precipitation index (SPI) was calculated for the years 1987–2011 using daily precipitation data from a meteorological station located within the study area. SPI has been used to evaluate the effects of precipitation patterns on the general availability of soil moisture for plants (i.e., wet or dry soil conditions) (Dutra et al, 2013). SPI has several advantages over other drought indices (e.g., Palmer Drought Severity Index) in that it is easy to calculate (only uses precipitation), easy to interpret (standardized value), and can be calculated across a range of temporal scales. SPI is a probability-based indicator that depicts the degree to which accumulative precipitation for a specific time period departs from the average state [51]. The SPI is a based on the cumulative probability of a given rainfall event occurring at a location. It is a spatially invariant indicator of abnormal wetness or dryness that explicitly accounts for the importance of time scales in the analysis of water availability and water use. Since the SPI is standardized, an index of zero indicates the median precipitation amount (i.e., normal conditions), while drought conditions and wet conditions are indicated by negative (i.e., exceptionally dry < = -2) and positive values (i.e., exceptionally wet > = 2), respectively. SPI can be calculated across a range of time scales, with short time scales (i.e., weeks), representing event-driven changes in water availability relating to dynamic ecosystem properties (e.g., annual grass emergence); and longer time scales (i.e., years) relating to the cumulative effects of prolonged drought or wetness (e.g., vegetation dieback or shifts in plant functional types). In arid regions with highly seasonal distributions of precipitation, the SPI calculated at very short time scales (<1 month) has been shown to produce unreliable results for characterizing abnormal dryness or wetness due to the high occurrence of no-rain cases which results in a highly skewed distribution [52]. Consequently, we selected time scales ranging from 1 to 36 months, to characterize both short-term event driven responses and longer-term precipitation anomaly effects.

## Results

### Ecological site model performance

The SVM model for ecological site classes had a 62 percent correct classification (PCC) and a QD and AD of 0.09 and 0.29, respectively (Table 2). The frequency distribution of ecological site classes was unbalanced in our study area, with 49% of sample locations classified as Sandy, 21% Loamy, 18% Deep sand, 9% Shallow sand, and 3% Clayey. In general, producer’s and user’s accuracies for individual ecological site classes were similar, indicating the model’s ability to both detect and predict sample classes with a similar level of accuracy (Table 3). An exception to this generalization was the Shallow sandy ecological class, where the model performed poorly in its ability to detect (producer’s accuracy) this class due to its tendency to misclassify it as the Sandy ES class. The ecological site classes with the lowest number of observations (i.e., Clayey and Shallow sandy) had the lowest accuracies, while the ecological site classes with the highest number of observations (i.e., Sandy and Loamy) had the highest accuracies.

**Table 2:** Model performance for SVM using leave-group-out cross validation and comparison to expert delineated and gSSURGO classified ecological sites.

	Ecological Site Classification		
	SVM	Expert	gSSURGO
PCC ^a^	62	89	51
95% CI	61–64	84–94	44–59
QD^a^	0.09	0.01	0.27
AD^a^	0.29	0.10	0.22

^a^ PCC, percent correctly classified; QD, quantity disagreement; AD, allocation disagreement.

**Table 3:** Error matrices for ecological site classes predicted using support vector machine (SVM) classification, expert delineation and gSSURGO classification.

		SVM						Expert Map						gSSURGO					
	Class ^a^	Reference						Reference						Reference					
		C	L	S	SS	DS	UA (%)	C	L	S	SS	DS	UA (%)	C	L	S	SS	DS	UA (%)
**Prediction**	**C**	41	30	0	0	0	58	5	0	0	0	0	100	0	0	0	0	0	0
	**L**	58	573	183	21	83	62	0	32	2	0	2	89	5	27	26	1	1	45
	**S**	1	353	1971	207	396	67	0	3	79	1	3	92	0	10	59	10	31	54
	**SS**	0	0	95	113	11	52	0	0	1	14	0	93	0	0	1	5	0	83
	**DS**	0	114	251	59	410	49	0	2	4	1	27	79	0	0	0	0	0	0
	**PA (%)**	41	54	79	28	46	**PCC = 62**%	100	86	92	88	84	**PCC = 89**%	0	73	69	31	0	**PCC = 51**%

^a^ C, Clayey; L, Loamy; S, Sandy; SS, Shallow sandy; DS, Deep sand; UA, user’s accuracy; PA, producer’s accuracy; PCC, percent correctly classified.

Ecological site classes at all 176 field locations were extracted from both gSSURGO dominant condition and expert delineated ecological site maps, and used to assess map accuracies. The gSSURGO ES map had a lower classification accuracy (PCC = 52%), while the expert delineated map had a higher classification accuracy (PCC = 89%) relative to the SVM model (Table 2). Error matrices for both gSSURGO and expert ES maps are shown in Table 3. The distribution of gSSURGO mapped ES classes displayed slightly different distributions relative to field identified values, with ESs more highly skewed towards the Sandy ES class and the omission of both the Clayey and Shallow sandy ES classes. In contrast, the ES class distribution for the expert map was similar to both field identified values and SVM predicted values. Total disagreement between validation and prediction datasets was primarily due to AD for the SVM predictions (AD = 0.29, QD = 0.09) and expert map (AD = 0.10, QD = 0.01), while gSSURGO showed similar level of disagreement due to both AD (0.22) and QD (0.27) (Table 2). Model misclassification represented by producer’s accuracies and the number of misclassified predictions occurring in each adjacent class for SVM predictions and both gSSURGO and expert ES maps are shown in Table 3.

Similar spatial patterns of mapped ES classes can be seen in both the SVM prediction map and the expert delineated ES map (Fig 4). Strong correspondence in spatial patterns can be seen for both spatially dominant (e.g., Sandy) and less common (e.g., Clayey) ES classes (Fig 4). The cumulative area for each ES class is also very similar between the SVM prediction map and the expert delineated ES map (S1 Fig). The lack of field sample points for the Gravelly loam, Drainage, Tank, and Road ES classes precluded them from our SVM prediction map, however, these classes only comprised ~1% of the expert delineated ES map. Spatial patterns of ES classes in the gSSURGO dominant condition map (Fig 3A) show that it effectively delineated the generalized area of the most dominant ESs, but fails to provide accurate information on their true spatial locations. When the gSSURGO maps of dominant (Fig 3A) and second most dominant (Fig 3B) condition are viewed together, we can see that it provides a rough indication of the spatial location of the four main ES classes in the study area, excluding the Clayey ES due to its minimal spatial coverage (~3%).

### Ecological states and model misclassification

**Fig 5:**
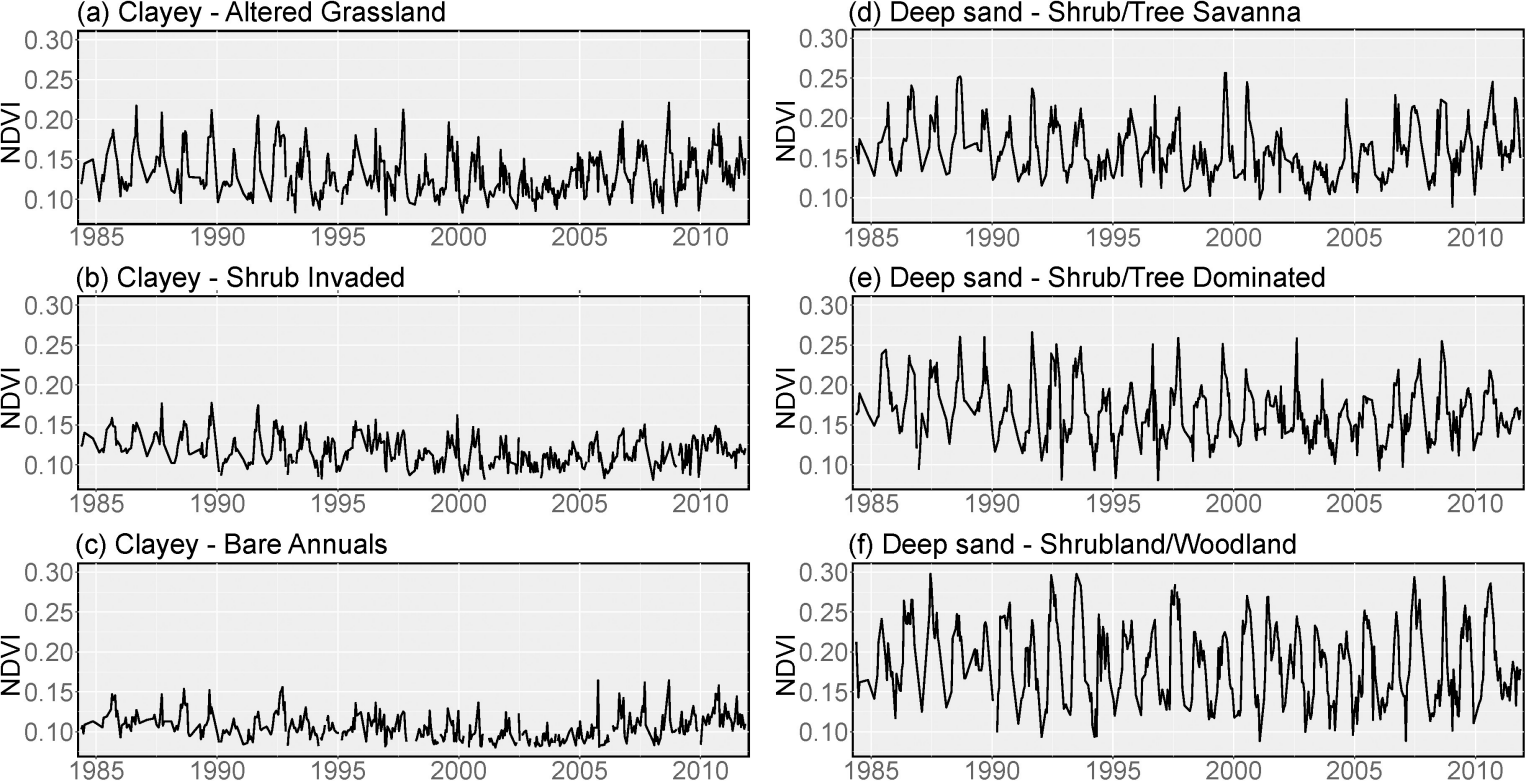
Examples of unfiltered NDVI hyper-temporal spectra from selected sampling points for the Clayey and Deep Sand ecological site classes. Clayey sites represent a sequence from least disturbed (a) to increasing levels of disturbance (b, c). Similarly, Deep Sand sites represent a sequence from least disturbed (d) to increasing levels of disturbance (e, f).

ESs can exist in several different stable ecological states (Table 1). These states can have highly contrasting vegetation communities that produce distinct spectral signatures. For example, Fig 5 illustrates three different stable ecological states for the Clayey (Fig 5A–5C) and Deep sand (Fig 5D–5F) ESs. Ecological state influences the spectral signature of both ESs, with decreasing amplitude for the Clayey ES as it transitions from an altered grassland (SC2) to a shrub-invaded (SC4) or bare/annuals (SC7) state. An opposite trend was seen for the Deep sand ES, where seasonal amplitudes increased from the shrub/tree savanna (SC3) state to the shrub/tree dominated (SC5) and shrubland/woodland (SC6) states.

**Fig 6:**
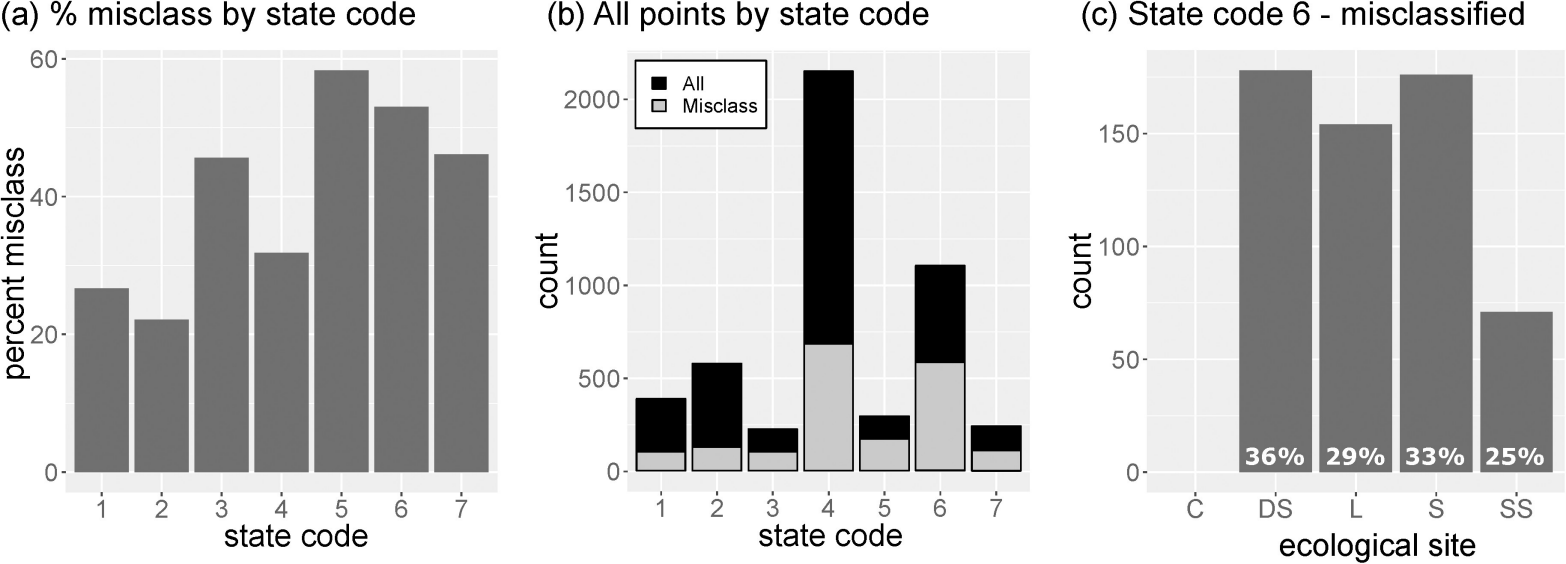
Barplots illustrating model misclassification by ecological site and state. (a) the percentage of misclassified points within each ecological state code; (b) the sample size distribution of all points (black) and all misclassified points (grey) broken out by state code; and (c) all misclassified points that had a shrub woodland state (state code 6) broken out by ecological site. Also, the percentage of misclassifications within each ecological site class that had a state code of 6 (c) is listed at the base of each bar.

In order to examine if certain states have a higher likelihood of being misclassified, model misclassification was evaluated relative to the ecological state of misclassified points. Results from this analysis showed that the percentage of misclassified points for each state code increased with increasing degree of disturbance (e.g., historical grassland: SC1-SC2-SC4-SC6-SC7, or historical savanna: SC3-SC5-SC6) (Fig 6A). Additionally, the less common ecological states within our sample dataset had a higher occurrence of being misclassified (e.g., SC3, SC5, SC7), with the exception of SC6 (shrub woodland) which was the second most common ecological state (Fig 6B). The contribution of each ecological site towards the number of misclassified points for state code 6 is shown in Fig 6C. The percentage of misclassified points for each ecological site that had a state code of 6 (Fig 6C) is also shown. For example, 36% of all misclassified Deep sand ESs had a state code of 6.

**Fig 7:**
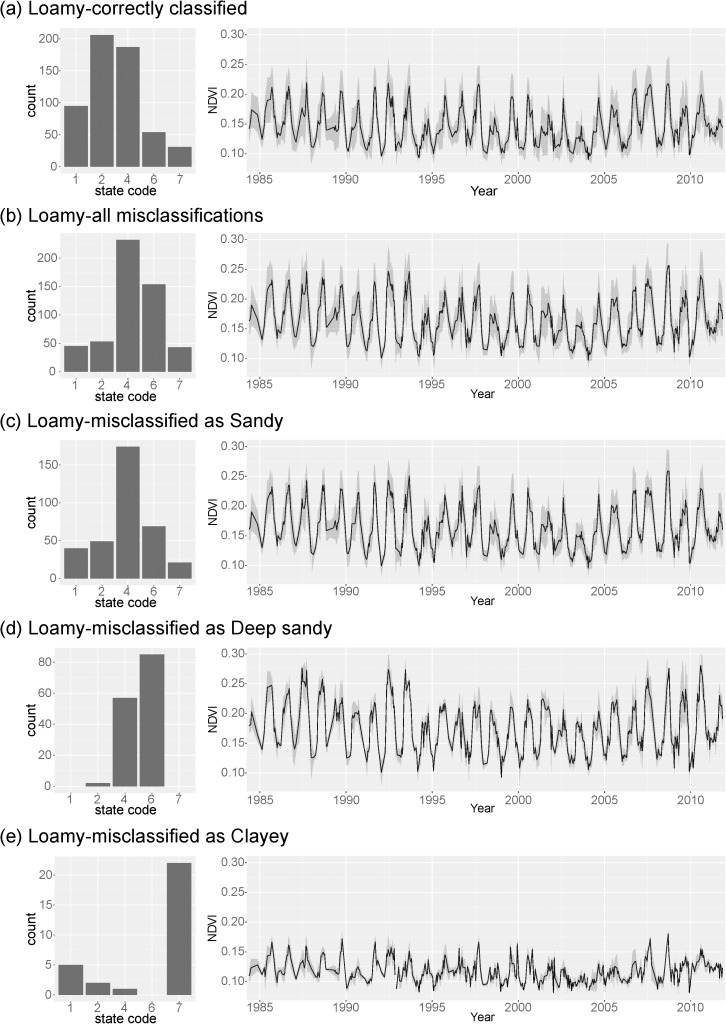
Results on the effect of ecological state on the misclassification of the Loamy ecological site. The frequency distribution of ecological states is shown for (a) all correctly classified Loamy sites and their corresponding spectral signature (mean +/- 1SD); (b) all misclassified Loamy sites and their corresponding spectral signature; (c) all Loamy sites misclassified as Sandy and their corresponding spectral signature; (d) all Loamy sites misclassified as Deep Sand and their corresponding spectral signature; and (e) all Loamy sites misclassified as Clayey and their corresponding spectral signature.

The effects of alternative states on ES misclassifications are further illustrated for the Loamy ES, where the state code designation for both the correctly classified and misclassified points and their corresponding spectral signatures (mean +/- 1 SD) are shown in Fig 7A and 7B. While the spectral signatures for the correctly classified and misclassified points appear similar, the misclassified points have a higher amplitude for most of the time-series. Misclassified values were further subdivided by the ES class they were incorrectly assigned, with its corresponding spectral signature and distribution of state codes for those incorrect designations presented in Fig 7C–7E. Correctly classified Loamy sites predominantly belonged to grassland (SC1), altered grassland (SC2), or shrub-invaded (SC4) state classes. In contrast, Loamy ES sampling points that were misclassified as Clayey were predominantly in the bare earth/annuals ecological state (SC7) and had a very low amplitude signal (Fig 7E) relative to the correctly classified Loamy sites (Fig 7A). This was in further contrast to the Loamy sites misclassified as Deep Sand, which were predominantly in the shrub woodland ecological state (SC6) and had a very high amplitude signal (Fig 7D) relative to the correctly classified Loamy sites.

### Precipitation-NDVI temporal dynamics and relationship to covariate importance

**Fig 8:**
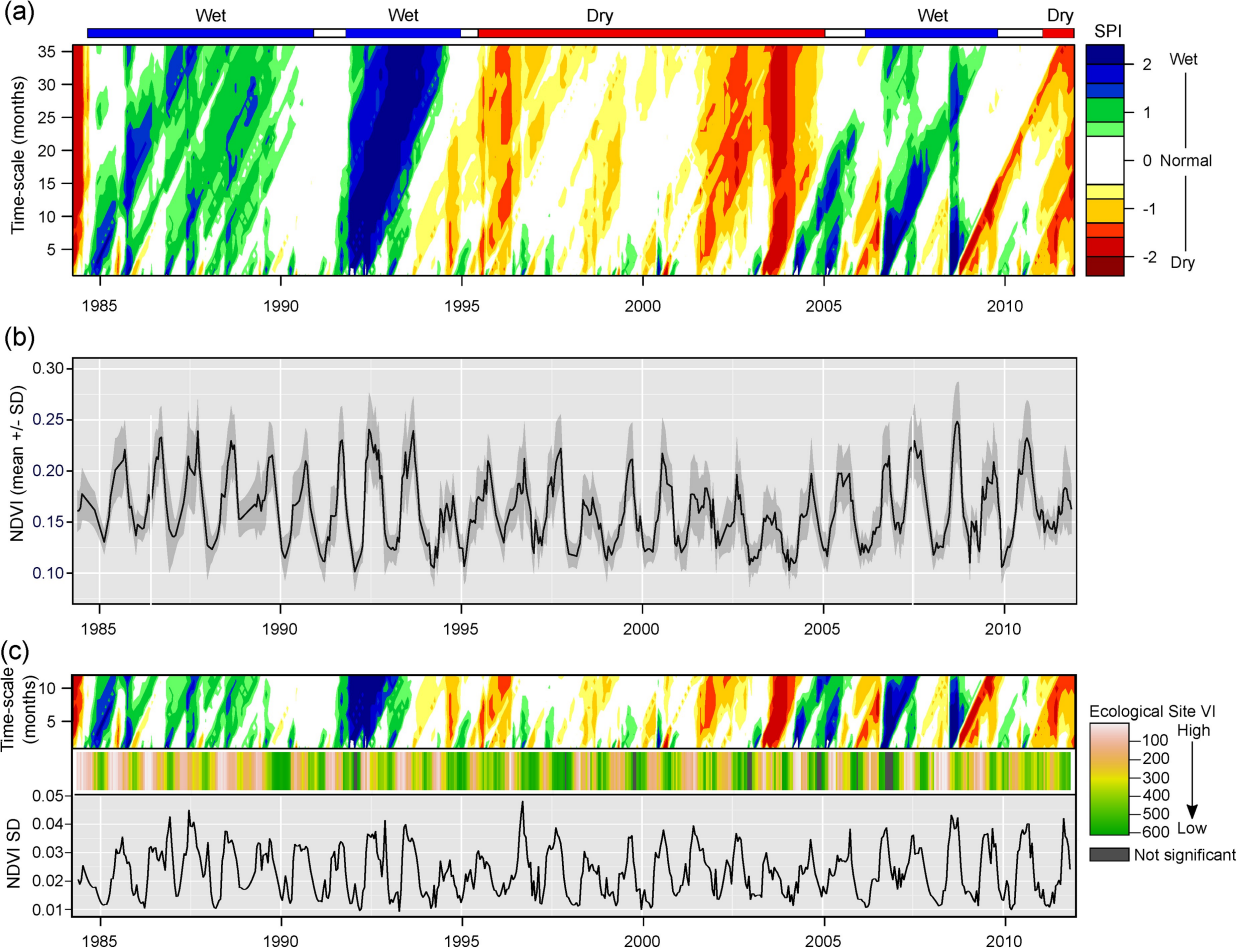
Temporal patterns in SPI and NDVI, and their relationship to SVM variable importance. (a) standardized precipitation index (SPI) calculated across a range of time scales illustrating general climatic states (e.g., wet, dry normal) at coarse temporal scales (~35 months) and event driven responses at short time-scales (~0–6 months); (b) mean +/- 1 standard deviation of NDVI calculated from Landsat pixels at all 176 sample locations; and (c) tri-panel illustrating relationship between SVM variable importance (VI) for ecological site prediction model (middle panel) and both short-term SPI (top panel) and NDVI standard deviation (bottom panel). See methods section for description of interpreting SPI time-scales.

Using the three SPI climatic categories (i.e., wet, dry normal), we identified four distinct climatic periods: a period of wet conditions (1984–1994), followed by dry conditions (1995–2004), followed by wet conditions (2005–2010), and then finally another period of dry conditions (2011+) (Fig 8A). Short periods of average precipitation were generally seen during transitions between wet or dry periods. Distinct and sporadic precipitation events at short time-scales (i.e., 1–6 months) resulted in highly dynamic fluctuations between abnormally wet and dry conditions, as shown by the high number of fluctuations in SPI along the time series at the shortest temporal scales (Fig 8A).

Variability in NDVI seasonal amplitude corresponded to precipitation patterns both at long and short time-scales. Mean NDVI for all 176 sample locations showed patterns in seasonal amplitude that generally corresponded to changes in climatic states at coarse temporal scales (Fig 8B). For example, during both of the wet climatic periods (1984–1994; 2005–2010), both the amplitude and maximum NDVI values were higher relative to the dry climatic periods (1995–2004; 2011). Variability in precipitation events at short time-scales also influenced variability in NDVI seasonal amplitude during the different climatic periods (Fig 8A). For example, a period of extreme dryness in the summer of 2003 contributed to a highly muted NDVI seasonal signal (Fig 5B). The standard deviation (SD) of NDVI at all 176 sample locations across the time series shows a seasonal pattern, with highest SD values occurring during periods of maximum NDVI (i.e., annual peak biomass), and lowest SD values occurring during periods of minimum NDVI (i.e., vegetative dormancy and senescence) (Fig 8C). All significant RS scenes with their corresponding ranked value were reordered by image date to provide a visual indication of which scenes along the time series contributed the most to model performance (Fig 8C). Evaluating the temporal sequence of covariate importance for the model within the context of short-term precipitation effects (1–6 month SPI) and NDVI variability (NDVI SD) revealed a distinct pattern where significant scenes within the model occurred after abrupt transitions between short-term climatic states (Fig 8C).

## Discussion

### Soil-vegetation response to climatic variability

Central to the concept of ESs is the tight coupling between potential vegetation composition and climo-edaphic properties. While a static inventory of vegetation composition can provide some insight into the potential distribution of ESs, considerable uncertainty remains due to the fact that similar vegetation communities can exist on contrasting soil types and ecological site classes, and alternative states can occur on the same soil types. Thus vegetation alone cannot adequately differentiate ESs, highlighting the need for additional information on the soil properties that regulate vegetation dynamics. Recent work [31] has demonstrated the utility of using hyper-temporal Landsat NDVI to predict soil properties through characterizing a spectral ‘fingerprint’ of the soil-vegetation relationship through time. Based upon this fundamental premise, our work utilized hyper-temporal NDVI time series to characterize the distinct vegetation-soil-climate relationships that differentiate ecological site classes at each pixel location across our study area.

Prior research has also shown that soil, and in particular soil moisture, exerts a dominant control on the spatial distribution and temporal dynamics of vegetation in semiarid landscapes [53–57]. Antecedent soil moisture has a demonstrated and pronounced effect on net primary production [58,59] and the resulting spectral response of vegetation [60]. The ability to accurately predict ecological site classes is dependent upon identifying periods of variability in NDVI spectra that correspond to the highest between class variation. In general, periods along the time-series associated with a high variable importance in the SVM model occurred during transitions between different climatic states (e.g., wet-to-dry) (Fig 8). Our results show that variability in the amplitude of inter-annual NDVI and NDVI SD at peak biomass is related to variability in short-term SPI which characterizes transitions between all types of climatic states (i.e., wet, normal, dry). Different ESs will have unique spectral responses to different climatic transitions depending on their unique assemblage of vegetation and soil properties. For example, a Clayey ecological site dominated by annual grasses will likely experience a more rapid greening response to an intense precipitation event relative to a Loamy ecological site dominated by perennial grasses [61,62]. The effect of long-term precipitation anomalies (i.e., SPI 20–36 months) amplifies these differences and drives the frequency of high variability transition events (Fig 8). For example, in the fall of 2004 a period of high rainfall corresponded to a series of scenes with the highest variable importance. This period of high rainfall directly followed a period of approximately 8 months of intense drought (detected at both short and long time-scales), preceded by approximately 10 years of moderate drought (Fig 8). ESs in degraded ecological states (e.g., shrub woodland, bare), however, exhibit spectral signals that are largely invariant to climatic transitions, thus complicating the ability to differentiate between ESs within these states (Figs 6 and 7).

### Model based mapping of ecological sites

The spatial prediction of ESs using hyper-temporal Landsat imagery was highly effective, producing both spatial and areal distributions that closely corresponded to the expert delineated map of ESs (Fig 4 and S1 Fig). Although our sample dataset included non-random points (rapid field traverses) which may have introduced bias into our estimates, the close correspondence between the SVM prediction and the expert map (a form of external validation) support the effectiveness of SVM in predicting ESs. Prediction accuracy for our SVM model was higher than gSSURGO but appreciably less than the expert map (Table 2). Given that gSSURGO is a map of the dominant ES class within each soil map unit, it is not surprising that it over predicts the dominant ES classes and fails to represent the rare ES classes (Clayey and Shallow sandy), as shown by its individual class accuracies (e.g., producer’s and user’s accuracies) (Tables 2 and 3). In addition, the high prediction accuracy for the expert map may in part be due to the fact that ES classes were delineated using the sample dataset and thus PCC is not a true estimation of map accuracy due to the lack of an independent validation dataset. Similar spatial patterns between the SVM model and the expert map suggest a similar ability to predict ESs, however, additional testing using an independent validation dataset will be needed to conclusively compare their performance. It is clear, however, that the high cost and time investments required for the expert mapping approach make it unfeasible for widespread adoption. In contrast, the hyper-temporal remote sensing approach provides a rapid and effective method of mapping ESs at a 30 m spatial resolution, providing needed information for land managers to implement appropriate management actions at the site, landscape, and regional scales.

The frequency distribution of ES classes was unbalanced for our sample dataset due to both the spatial distribution of ES classes within our study area and the sampling strategy used (i.e., random and purposive sampling). The study area has a high degree of heterogeneity in biogeomorphic properties (e.g., vegetation, soils, topography), resulting in a highly uneven spatial distribution of sample classes. In addition, the sample dataset used in this study was a collection of four separate field sampling campaigns rather than a single targeted sample design. The collection of sampling points using a probabilistic design is critical to ensure that the sample is representative of the larger population, thus minimizing bias and maximizing confidence in model estimates. In future efforts, a targeted probabilistic sampling design stratified by the hyper-temporal NDVI image time series (e.g., hierarchical clustering, conditioned latin hypercube sampling) would likely provide a more balanced sample distribution among classes, resulting in a more accurate classification of ESs.

An additional factor that can impact model accuracy is the potential for inaccuracies within the point data used to train and validate the SVM model. Although our aggregated dataset employed similar vegetation and qualitative soil surface sampling protocols, they differed widely in their method of soil sampling. As has been previously discussed, accurate information on relevant soil properties is critical in delineating ES classes. We evaluated the internal accuracy of each dataset and examined these accuracies with respect to the soil sampling method employed. A general trend of increasing model accuracy is seen in the datasets that characterized the soil more thoroughly, with 46% misclassification in the dataset that employed selective soil sampling (SB), 28 and 34% misclassification in the datasets that employed soil augering (DG and TR, respectively), and 13% misclassification in the dataset that employed soil mini-pits (MV) (S2A Fig). It should be noted that other differences between the datasets limits their direct comparability, such as differences in sample size and ES class distribution (S2B Fig), however, these results suggest the importance of accurate soil characterization for predicting ES classes (see SI section for additional information on sample datasets).

Finally, model accuracy can be negatively impacted by RS sensor noise and errors in the geographic registration of RS imagery and sampling points, which may result in the incorrect prediction of ESs at individual pixels. Applying spatial filters (e.g., 3 x 3 moving window) that match the scale of the MMU associated with field sampling can help to remove misclassifications resulting from sensor noise and registration errors. In this study, our use of leave-group-out cross validation and our lack of an independent validation dataset prevented us from being able to evaluate the effects of spatial filtering on model accuracy.

### Evaluating ecological site misclassification

Hyper-temporal remote sensing is highly effective in mapping ESs due to the fact that it characterizes temporal variability in vegetation spectral response which is driven by climatic variability but modulated by soil type. In other words, during a normal year the alternative state of an ecological site (e.g., degraded loamy) may exhibit a phenology that more closely resembles a different ecological site (e.g., sandy) relative to its reference state. Yet, over time its spectral signal will fluctuate in response to climatic variability, and in particular climatic extremes (i.e., drought, abnormal wetness), producing a spectral signature that more closely resembles the reference state due to the controlling effect of soil type in modulating vegetation spectral response. Thus, hyper-temporal remote sensing is unique in its ability to quantify the spectral properties of each pixel location at a high temporal frequency, allowing the detection and characterization of a spectral ‘fingerprint’ capable of differentiating ESs.

While the effects of vegetation on NDVI spectral response has been well documented, the compounding effect of soil quality (e.g., soil fertility, erosion) on NDVI spectra, and in particular during climatic extremes is less known. Ecological sites can exist in a range of ecological states with highly contrasting vegetation communities, soil resource conditions and supported ecosystem services. Thus, a given ES can a have vastly different spectral response depending on its current state (e.g., Clayey and Deep Sand, Fig 5). As a result, the relationship between ESs and hyper-temporal spectral response functions is complicated by both within-class variation (alternative states) and between-class similarities. For example, the Loamy sites that were misclassified as Clayey had spectral signals with highly muted seasonal amplitudes similar to the Clayey ES class (Figs 5A–5C and 7E). Similarly, the Loamy sites that were misclassified as Deep Sand had spectral signals with high seasonal amplitudes similar to the Deep Sand ES class (Figs 5D–5E and 7d). These degraded states (e.g., shrub woodland, bare) produce highly stable spectral signals (i.e., invariant to climatic fluctuations) and thus the ESs in these states are more subject to misclassification (e.g., SC6, Fig 6A and 6C). Consequently, this study highlights an important factor that can impair the performance of the hyper-temporal approach, namely the under sampling of ES classes, and in particular the spectral endmembers that represent the within class variability (i.e., states).

While misclassification of ESs due to spectral similarity can result from an under sampling of the within-class spectral variability (i.e., characterizing the spectral response of alternative stable states), it can also be indicative of deficiencies in our ES concepts. The existing framework for ESD development is a correlative process, where soil series phases are grouped based on a similar vegetation, climate, and geomorphology that imparts a similar ecological potential [15]. This process employs both expert knowledge and quantitative methods such as high intensity sampling and data driven analytics (e.g., multivariate clustering) to describe and delineate ESs. The processes that led to the observed patterns are then inferred based on first principles and expert knowledge. This approach, however, is highly subjective and can result in identified processes that do not directly account for the temporal dynamics that gave rise to the observed patterns. In other words, in many cases it can be unclear whether the ES concepts themselves are valid or just an artifact of landscape patterning. For example, recent discussions amongst local experts have brought into question whether the Shallow sandy ES class is sufficiently unique relative to the Sandy ES class to warrant its own class designation. Results from this study showed that the Shallow sandy ES class had a substantially lower producer’s accuracy relative to other classes and a high rate of misclassification as the Sandy ES class, thus supporting the reevaluation of the Shallow sandy ESD concept. The implication of this result is that our model accuracies may in fact be higher than reported because of inconsistencies in the ES concept definitions. Consequently, the hyper-temporal RS modeling framework may also provide an approach to test and refine the concepts underpinning our ESDs from first principles, through quantifying the temporal dynamics of vegetation which are largely modulated by soil properties.

### Future considerations for mapping ecological sites

Current efforts to create detailed maps of ESs in the U.S. are working within the existing framework of NRCS SSURGO soil map units. While this study provides a comparison to this approach, our mapping framework is independent of existing mapping systems and thus has the potential of being applied anywhere in the world given the availability of RS imagery and point data to train the classification model. In theory, the hyper-temporal remote sensing approach should work in any ecosystem where individual landscape units, comprised of unique assemblages of vegetation and soil properties, exhibit unique spectral responses to different climatic transitions. Future research is needed to examine NDVI temporal patterns in other ecosystem types (e.g., forests, wetlands) and whether these patterns can be used to differentiate ecological sites. Consideration also needs to be given regarding the quality of RS imagery, particularly in areas prone to extended cloud cover or other sources of environmental interference that may negatively impact the signal-to-noise ratio. Additionally, the high occurrence of misclassification for ES classes in degraded ecological states (e.g., shrub woodland, bare) highlights the need for greater sampling of spectral endmembers to better characterize the within class spectral variability. Improved sampling designs randomly stratified across covariate space (e.g., hyper-temporal image stack), combined with standardized soil sampling protocols that fully describe the soil profile (i.e., mini-pits), will help to better characterize the within class spectral variability and ultimately improve ES predictions.

The identification of additional environmental covariates may also help to further differentiate ES classes. For example, incorporating terrain attributes (e.g., slope, aspect, curvature, topographic wetness index) may assist in better discriminating between ES classes that have similar spectral properties because different ES classes typically occupy different landscape positions and are characterized by different terrain features [16,55].

Future work is needed to examine the ability to scale this approach to the regional scale with the possible use of MODIS 250-m imagery, as recent research at the JER has shown that MODIS provides a higher signal-noise ratio relative to Landsat while still providing comparable information in terms of ecosystem structure and change [63]. It is recognized that these scaling relationships will change in different ecosystem types and additional research is needed to test this approach in other ecosystems that experience different natural and anthropogenic drivers.

## Conclusions

The development of ESs as management units coupled with STMs has emerged as a highly effective land management framework, but its utility has been limited by spatial ambiguity of ES locations in the U.S., lack of ES concepts in many other parts of the world, and the inability to accurately assess the ecological state of ESs or monitor changes in state through time. These constraints present many challenges in light of the rate and extent of change occurring across both natural and managed ecosystems in response to a wide range of natural and anthropogenic drivers. Consequently, new approaches and techniques are being sought that can provide spatially explicit ES information required for effective land management.

In response to this need, we have presented a spatially-explicit modeling framework for predicting the location of ESs in a semi-arid ecosystem. We have demonstrated that a hyper-temporal remote sensing approach is effective in characterizing the soil-vegetation relationship and its response to climatic variability. Furthermore, we showed that high spectral variability in the NDVI time-series, resulting from transitions between climatic states, corresponded to NDVI scenes with the highest importance in our SMV model, confirming the importance of spectral variability in predicting ES classes. Consequently, the optimal time-series for modeling ES classes is not dependent upon its length, but rather that it encompasses a time period that includes a range of climatic extremes which result in maximized spectral variability. However, given spectral similarities observed between ES classes with high occurrences of misclassifications (e.g., shrub-invaded, shrub-dominated), three considerations need to be addressed to improve model predictions: 1) sampling designs must represent the range of both within class (i.e., states) and between class (i.e., ESs) spectral variability by sampling across covariate space, 2) soil sampling protocols need to provide a thorough and accurate characterization of key soil properties (e.g., texture, depth), and 3) additional environmental covariates need to be evaluated that may help further differentiate sites with similar spectral signals.

Finally, the proposed hyper-temporal RS technique may provide an objective framework to evaluate and test ES concepts, examining differences in vegetation dynamics in response to climatic variability and other types of land-use change. Given limited financial and human resources, an improved understanding of ecosystem potential is needed to maximize ecosystem services, promote the recovery of degraded lands, and adapt to and mitigate the impacts of climate change [64]. The hyper-temporal remote sensing approach presented here has potential to greatly improve the efficiency of high-resolution ecological site mapping; however, additional research is needed to test the efficacy of the proposed approach in other ecosystems with different underlying change drivers and environmental controls on ecosystem structure and function.

## Supporting information

S1 Fig Cumulative area for each ecological site class within, (a) expert delineated ecological site map, and (b) SVM predicted ecological site map.
(TIF)

S2 Fig The misclassification rate and the distribution of ecological sites for the four sample datasets. (a) the percentage of misclassified sample points from each soil sampling campaign used in our combined sample dataset, and (b) the sample size and distribution of ecological sites within each dataset. The soil sampling method employed for each individual dataset is show in (a). The SVM model error rate using all four datasets was 38%.
(TIF)

S1 Table Descriptions of dominant ecological sites within the study area. (DOCX)
