# Supplementary figures and images for: A hyper-temporal remote sensing protocol for high-resolution mapping of ecological sites
Source: PLoS One. 2017 Apr 17;12(4):e0175201. doi: 10.1371/journal.pone.0175201 (PMC5393606; doi:10.1371/journal.pone.0175201)

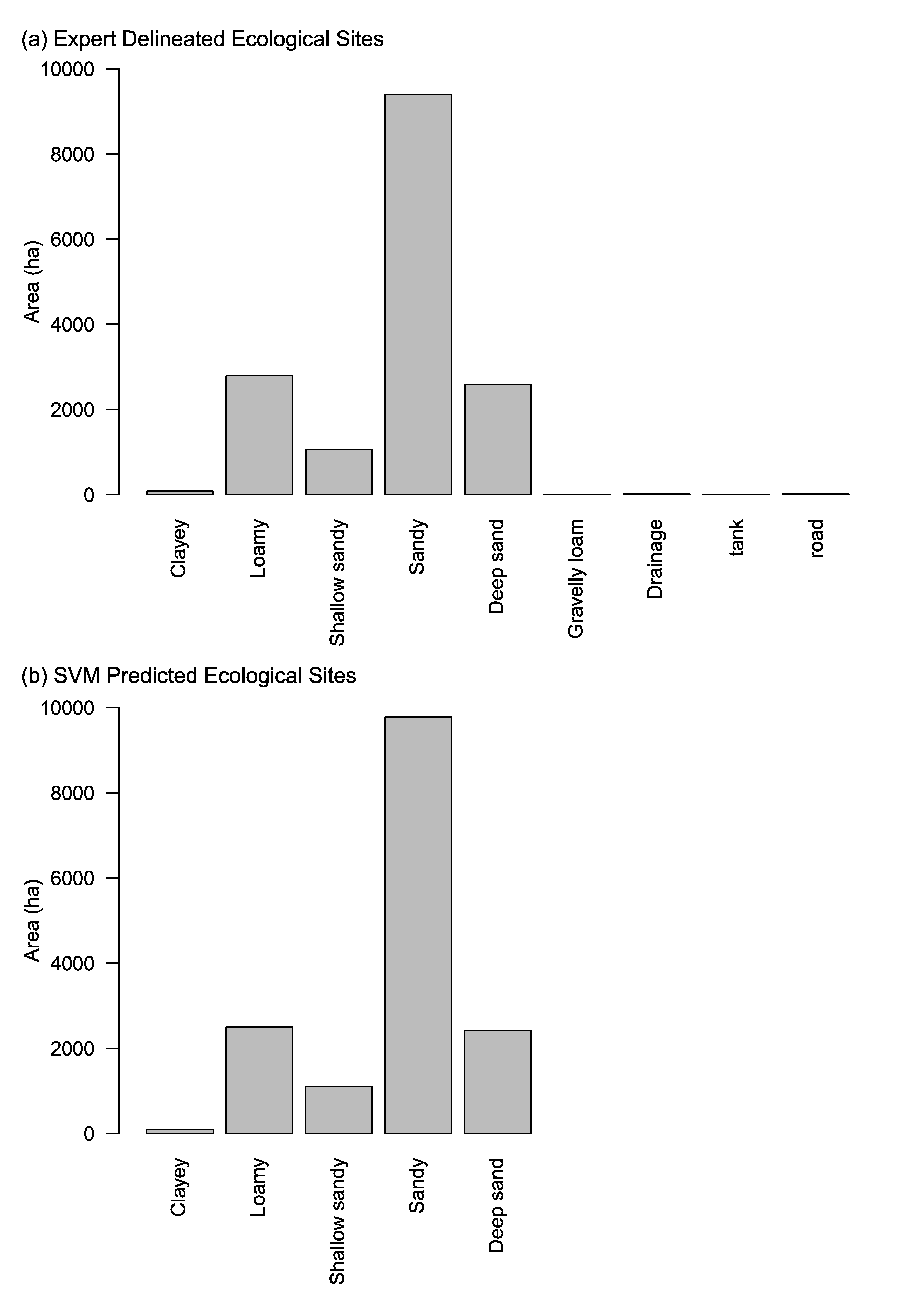

Supplement: S1 Fig — Cumulative area for each ecological site class within, (a) expert delineated ecological site map, and (b) SVM predicted ecological site map. (TIF) [file pone.0175201.s001.tif]

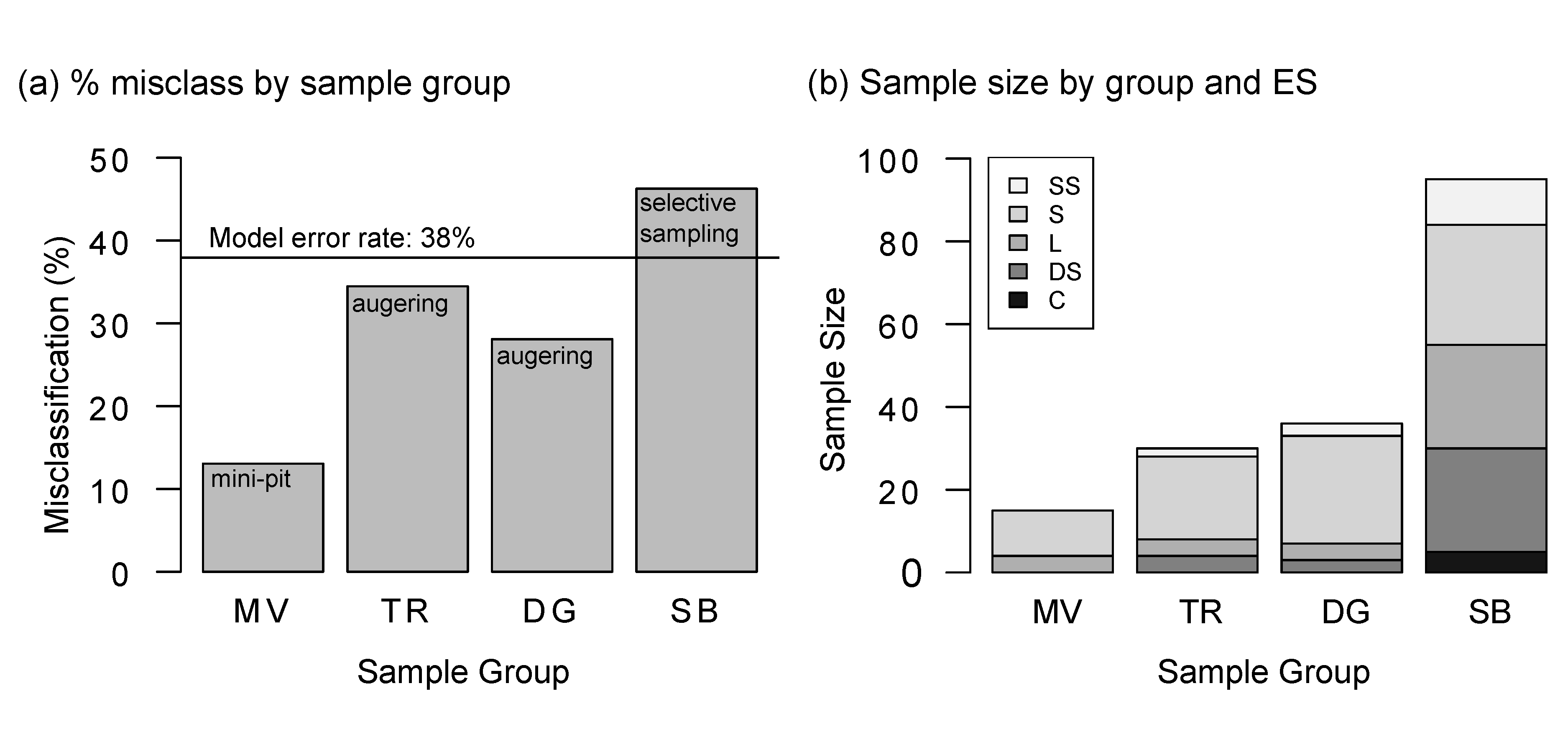

Supplement: S2 Fig — (a) the percentage of misclassified sample points from each soil sampling campaign used in our combined sample dataset, and (b) the sample size and distribution of ecological sites within each dataset. The soil sampling method employed for each individual dataset is show in (a). The SVM model error rate using all four datasets was 38%. (TIF) [file pone.0175201.s002.tif]
