# Supplementary material for: A hyper-temporal remote sensing protocol for high-resolution mapping of ecological sites
Source: PLoS One. 2017 Apr 17;12(4):e0175201. doi: 10.1371/journal.pone.0175201 (PMC5393606; doi:10.1371/journal.pone.0175201)
Supplement: S1 Table — (DOCX) [file pone.0175201.s003.docx]

S1 Table. Descriptions of dominant ecological sites within the study area

| Ecological Site | Description† |
| --- | --- |
| **Clayey** | **Soil/geomorphology**: This site typically occurs on piedmont slopes, alluvial flats, or permanently drained floodplains. Slopes are usually flat, averaging less than 5%. Parent material consists of slightly-to-moderately calcareous mixed alluvial sediments derived from sedimentary material.  **Vegetation**: Historical plant community is dominated by tobosa (*Pleuraphis mutica*) and to a lesser extent by black grama (*Bouteloua eriopoda*).  **State Changes**: Dominant change drivers include grazing and drought which affect patterns of soil water availability, changes to soil structure, and soil truncation. The establishment of burrograss (*Scleropogon brevifolius*) increases in response to grazing and drought. Shrub encroachment by a variety of species, including tarbush (*Flourensia cernua*), mesquite (*Prosopis glandulosa*) and creosotebush (*Larrea tridentata*) occurs within this site as bare patches increase. Shrub dominance and grass loss is associated with truncation of the A horizon and sealing of the eroded soil surface. Subsequent loss of shrubs or continued soil degradation in the absence of shrub seeds may produce a non-vegetated clay flat, which may accumulate salts or carbonate at the surface. |
| **Loamy** | **Soil/geomorphology**: This site typically occurs on level to gently sloping fan piedmont, alluvial fans, fan remnants or flood plains. Soils are derived from igneous and sedimentary sources.  **Vegetation**: Historical plant community is dominated by black grama and tobosa, with lesser amounts of aalkali sacaton (*Sporobolus airoides*).  **State Changes**: Dominant change drivers include selective herbivory and drought which promote erosion and soil truncation. Increasing dominance of burrograss, tobosa, or threeawns (*Aristida spp.*) may occur in response to grazing. Shrub encroachment by tarbush, honey mesquite and creosotebush occurs in bare areas where grass competition and/or fire is diminished. A shrubland state (often dominated by tarbush or creosotebush) with little grass cover eventually occurs due to loss of remaining grass cover, erosion, and soil truncation. |
| **Sandy** | **Soil/geomorphology**: This site typically occurs on level to gently sloping or undulating piedmont slopes, fan piedmonts, alluvial fan, fan remnant, dunes or plains. This site usually exists as a finely-scaled mosaic with the Shallow sandy ES depending on local variation to the depth of indurated caliche (petrocalcic horizon).  **Vegetation**: Historical plant community is dominated by dominated by black grama and other grasses, especially dropseeds (*Sporobolus spp*.).  **State Changes**: Dominant change drivers include overgrazing and prolonged periods of spring/summer drought or shrub invasion via introduction of mesquite seeds. Continuous heavy grazing results in a steady decline in black grama, followed by dropseed, which can lead to the persistent absence of these species (annual/bare state) or the invasion of shrub species. Subsequent grazing by livestock and native herbivores, competition from shrubs, erosion, and concentration of nutrients under adult shrubs eventually leads to persistent reductions of grass cover and mesquite-dominated coppice dunes with bare or snakeweed dominated interdunal areas. |
| **Shallow Sandy** | **Soil/geomorphology**: This site typically occurs on gently sloping to undulating hill slopes, fan piedmonts, basin floors, fan remnants and fan piedmonts. This site usually exists as a finely-scaled mosaic with the Sandy ES depending on local variation to the depth of indurated caliche (petrocalcic horizon).  **Vegetation**: Historical plant community is dominated by black grama and other grasses, especially dropseeds. The Shallow sandy site differs from the Sandy site in the greater production and dominance of black grama compared to other grass species in the historic community.  **State Changes**: Dominant change drivers include overgrazing and/or multi-year periods of summer drought, as well as the introduction of honey mesquite seeds with or without grazing. The nature of the states, communities, and transitions are nearly identical to the Sandy site, however, the probability of transitions to degraded states may be lower in the Shallow sandy site due to the role of the petrocalcic horizon in maintaining water at the grass rooting depth and physically impeding the progress of deeper shrub roots. |
| **Deep Sand** | **Soil/geomorphology**: This site typically occurs on level to gently sloping old eolian and alluvial deposits Parent material consists of eolian deposits and alluvium derived from sandstone. This site often intergrades with the Sandy ES.  **Vegetation**: Historical plant community is primarily dominated by by dropseeds (Sporobolus flexuosus, S. contractus, S. cryptandrus), especially the giant dropseed (S. giganteus). Black grama (Bouteloua eriopoda) and bush muhly (Muhlenbergia porteri) are also important grasses.  **State Changes**: Dominant change drivers include excessive grazing and drought. With increasing grazing pressure, black grama and bush muhly decline, and eventually dropseeds decline. Common shrubs species at the site include sand sage (*Artemisia filifolia*) and broom dalea (*Psorothamnus scoparius*), which increase in representation and cover with grazing and/or drought. |

†Ecological site descriptions were adapted from official ESD: https://esis.sc.egov.usda.gov/
